# First cytogenetic analysis of lesser gymnures (Mammalia, Galericidae, *Hylomys*) from Vietnam

**DOI:** 10.3897/CompCytogen.v12i3.27207

**Published:** 2018-08-23

**Authors:** Svetlana V. Pavlova, Larisa S. Biltueva, Svetlana A. Romanenko, Natalya A. Lemskaya, Anton V. Shchinov, Alexei V. Abramov, Viatcheslav V. Rozhnov

**Affiliations:** 1 A.N. Severtsov Institute of Ecology and Evolution, Russian Academy of Sciences, 33 Leninsky prosp., Moscow 119071, Russia A.N. Severtsov Institute of Ecology and Evolution, Russian Academy of Sciences Moscow Russia; 2 Institute of Molecular and Cellular Biology, Siberian Branch of the Russian Academy of Sciences, 8/2 Acad. Lavrentiev ave., Novosibirsk 630090, Russia Institute of Molecular and Cellular Biology, Siberian Branch of the Russian Academy of Sciences Novosibirsk Russia; 3 Novosibirsk State University, 2 Pirogova str., Novosibirsk 630090, Russia Novosibirsk State University Novosibirsk Russia; 4 Zoological Institute, Russian Academy of Sciences, 1 Universitetskaya nab., Saint Petersburg 199034, Russia Zoological Institute, Russian Academy of Sciences Saint Petersburg Russia; 5 National Research Tomsk State University, 36 Lenin Avenue, Tomsk 634050, Russia National Research Tomsk State University Tomsk Russia

**Keywords:** cell culture, cryoconservation, FISH, insectivorous mammals, karyotype, telomeric sequence

## Abstract

Gymnures are an ancient group of small insectivorous mammals and are characterized by a controversial taxonomic status and the lack of a description of karyotypes for certain species. In this study, conventional cytogenetic techniques (Giemsa, CBG- and GTG-banding, Ag-NOR), CMA_3_-DAPI staining, and fluorescent *in situ* hybridization (FISH) with telomeric DNA probes were used to examine for the first time the karyotypes of lesser gymnures of group *Hylomyssuillus* Müller, 1840 from northern and southern Vietnam. All studied specimens had karyotypes with 2*n*=48, NF*a*=64. C-positive heterochromatic blocks existed in centromeric regions of 7 bi-armed autosomes and the submetacentric X chromosome. The Y chromosome is a C-positive and dot-like. The nucleolus organizer regions resided terminally on the short arms of 2 small bi-armed pairs. Positive signals at the telomeres of all chromosomes were revealed by FISH. CMA_3_-positive blocks were localized on the telomeric and pericentric regions of most bi-armed and acrocentric chromosomes. Despite the large genetic distances between *Hylomys* Müller, 1840, lesser gymnures from *H.suillus*-group from northern and southern Vietnam have similar karyotypic characteristics.

## Introduction

The order Erinaceomorpha is a diverse group of small insectivorous mammals that are widely distributed throughout Africa, Europe, and Asia. According to most current taxonomic systems, this order contains the single family Erinaceidae with 2 subfamilies: Erinaceinae (hedgehogs) and Galericinae (gymnures) (McKenna and Bell 1997, Hutterer 2005). However, their ancient origin, deep genetic divergence, and high morphological differentiation suggest that these 2 taxa should be ranked as families (Bannikova et al. 2014). Based on the latest multigene study, we consider gymnures to be representatives of a separate family, Galericidae.

The family Galericidae comprises 6 recent genera, with 6–12 species in total (Hutterer 2005, Bannikova et al. 2014). Karyotypes of Galericidae have been poorly studied (O’Brien et al. 2006) – only 3 gymnure species have been karyotyped: Mindanao gymnure (*Podogymnuratruei* Mearns, 1905), endemic to Mindanao Island, Philippines, with 2*n*=40, NF=76 (Rickart 2003); shrew gymnure (*Neotetracussinensis* Trouessart, 1909) from southern China, with 2*n*=32, NF=52 (Ye et al. 2006); and Hainan gymnure (*Neohylomyshainanensis* Shaw et Wong, 1959), endemic to Hainan Island, with 2*n*=32, NF=64 (Li et al. 2008). The karyotypes for lesser gymnures of the genus *Hylomys* Müller, 1840 remain unknown.

Lesser gymnures *Hylomys* spp. inhabit the Greater Sunda Islands, Indochina, and southern China (Hutterer 2005). In most current taxonomic systems, *Hylomys* s.str. contains 2 species: *H.parvus* Robinson et Kloss, 1916 is restricted to the highlands of Sumatra, and *H.suillus* Müller, 1840 is distributed throughout continental southeast Asia and the Sunda Islands (Frost et al. 1991, Corbet and Hill 1992, Ruedi and Fumagalli 1996, Hutterer 2005). According to current taxonomy, there are 7 subspecies (*H.s.suillus*, *H.s.dorsalis*, *H.s.maxi*, *H.s.microtinus*, *H.s.pequnensis*, *H.s.siamensis*, and *H.s.tionis*) of *H.suillus* (Hutterer 2005). A recent mtDNA analysis suggested that the taxon *H.suillus* (sensu Hutterer 2005) represents a paraphyletic association of 5 to 7 full species, including an undescribed taxon from southern Vietnam: *Hylomys* sp. (Bannikova et al. 2014). These authors also suggested that the name *Hylomyssuillus* should be applied only to the Java population, whereas the lesser gymnures from northern Vietnam could be treated as distinct species: *Hylomysmicrotinus* Thomas, 1925 (Bannikova et al. 2014).

In this report, we characterized for the first time the karyotypes of lesser gymnures of *Hylomyssuillus*-group from northern and southern Vietnam using a set of cytogenetic tools. Prior to comprehensive taxonomic revision of the group *H.suillus*, we use the name *Hylomyssuillusmicrotinus* for the gymnures from northern Vietnam and *Hylomys* sp. for those from southern Vietnam (see Bannikova et al. 2014).

## Material and methods

### Specimens

Gymnures were collected during biodiversity surveys carried out by the Joint Vietnam-Russian Tropical Research and Technological Centre in 2013–2014. Voucher specimens are deposited in the Zoological Museum of Moscow State University (ZMMU), Moscow, Russia and the Zoological Institute of the Russian Academy of Sciences (ZIN), Saint Petersburg, Russia. Two specimens (male ZMMU S-193936 and female ZMMU S-199642) from Northern Vietnam, Phu Tho Province, Xuan Son National Park (21°08'12"N, 104°56'11"E), and one specimen (female ZIN 101915) from Southern Vietnam, Dak Lak Province, Chu Yang Sin National Park (12°25'26"N, 108°21'52"E) were karyotyped. The animals were caught alive using locally made cage traps (Abramov et al. 2008).

### Cell cultures, preservation of cells, and chromosome preparations

Primary fibroblast cell cultures that were derived from tail biopsies of individuals from northern Vietnam were established and subsequently deposited to the cell banks of 2 cytogenetic laboratories (in Moscow and Novosibirsk, Russian Academy of Sciences). The cell cultures were established in parallel to prevent the loss of valuable material. As a result, the cell culture from a female (ZMMU S-199642) was deposited only to Moscow lab, whereas the Novosibirsk lab established the fibroblast cultures from a male (ZMMU S-193936) and female (ZMMU S-199642).

Each lab modified the standard cell culture protocol (Freshney 2010). Briefly, small pieces of tails were cultured in DMEM or αMEM (Invitrogen) that was supplemented with embryonic bovine serum (10% or 15%, respectively) with penicillin/streptomycin (5000 units/5 mg/ml or 10^5^ U/L/100 mg/L, respectively) and amphotericin B (2.5 mg/L) at 37°C and 5% CO_2_ for 3-4 weeks. In all cases, the cells were cryopreserved using a standard technique for mammalian fibroblast cell cultures, in which the cells were suspended in medium supplemented with a high concentration of serum (>40%) with a cryoprotectant, dimethyl sulfoxide (DMSO) (to a final concentration of 10%). Cryovials were kept in a freezer (-70°C) overnight and then transferred in an ultra-low-temperature container with liquid nitrogen for long-term storage.

Metaphase chromosome preparations from primary fibroblast cultures were made following the standard technique (Freshney 2010).

The standard field procedure for bone marrow cultures was used to obtain chromosome preparations for a female (ZIN 101915) from southern Vietnam.

### Chromosome staining and microscope analysis

Air-dried chromosome spreads of all specimens were stained conventionally with 2% Giemsa for 4-5 minutes and then submitted to differential staining.

To determine the location of heterochromatin, C-banding was performed per the standard technique (Sumner 1972) with some modifications, as described in Gladkikh et al. (2016).

The fluorochromes chromomycin A3 (CMA_3_) and 4,6-diamidino-2-phenylindole (DAPI) were applied to identify GC- and AT-rich heterochromatic regions, respectively (Lemskaya et al. unpubl.).

The standard trypsin-Giemsa staining technique (Graphodatsky and Radjabli 1988) with some modifications was used to identify homologies by G-bands. Chromosome spreads were treated with 0.25% trypsin solution (Paneco, Russia) at 25-30°C for 15-20 seconds, rinsed in 2xSSC buffer, and then stained with 2% Giemsa for 2-3 min.

Nuclear organizer regions (NORs) were detected by silver nitrate staining following Graphodatsky and Radjabli (1988).

To detect telomeric repeats, the G-banded metaphase chromosomes of a female from northern Vietnam was hybridized *in situ* with a fluorescein-conjugated peptide nucleic acid (PNA) probe from the Telomere PNA FISH Kit/FITC (K5325 from Dako, Glostrup, Denmark) following the manufacturer’s instructions.

Images were captured with a ProgRes CCD (Jenoptic) camera mounted on an Axioscope 2 plus (Zeiss) microscope with filter sets for DAPI, FITC, and rhodamine, using VideoTesT-FISH 2.0 and VideoTesT-Karyo 3.1. (VideoTesT, Saint Petersburg, Russia) software. A Leica DFC-295 CCD camera mounted on a DM1000 (Leica) or Metasystems CCD (Zeiss) camera mounted on an Axioscope 2 (Zeiss) microscope were used to capture all other non-fluorescence images using a Metasystems Ikaros ver.5.3 and Leica Application ver.3.2 softwares, respectively.

## Results

### Karyotypes of gymnures from northern Vietnam (*H.suillusmicrotinus*)

**Figure 1.**
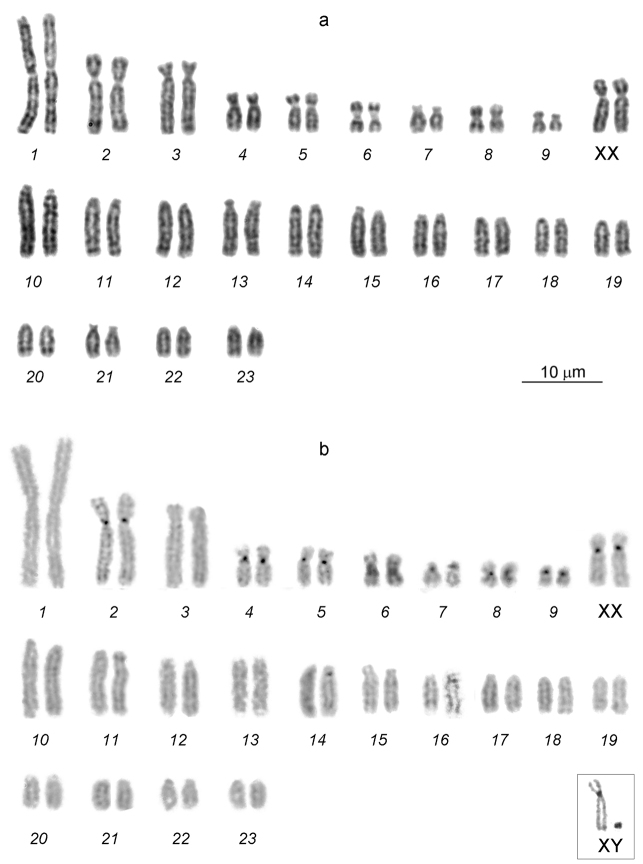
A female karyotype of the lesser gymnure *H.suillusmicrotinus* from northern Vietnam: conventional staining **(a)** and C-banding **(b)**. 2*n*=48, NF*a*=64. XX – the female sex chromosomes. C-banded sex chromosomes of a male (XY) are given in a frame.

**Figure 2.**
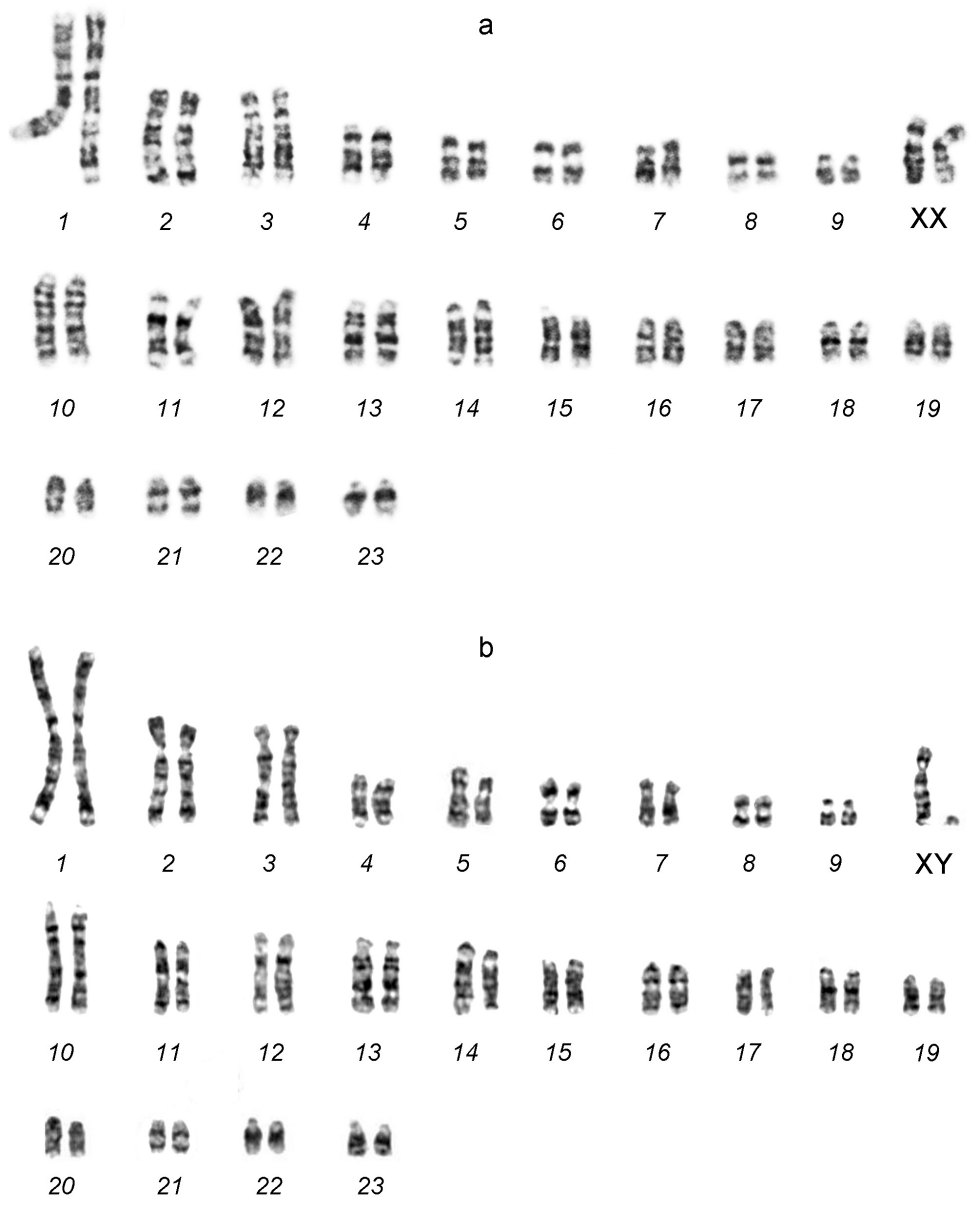
G-banded female **(a)** and male **(b)** karyotypes of the lesser gymnure *H.suillusmicrotinus* from northern Vietnam.

The diploid chromosome number of the male and female karyotypes was 2*n*=48, NF*a*=64 (Fig. 1a). The chromosome set consists of 10 pairs of bi-armed chromosomes and 14 pairs of acrocentrics. A pair of the largest metacentrics (№ 1), 2 pairs of large submetacentrics (№ 2–3), 2 pairs of medium-sized submetacentrics (№ 4–5), 2 pairs of medium-sized metacentrics (№ 6, 8), 2 pairs of small submetacentrics (№ 7, 9), and large-to-small acrocentrics (№ 10-23) represent an autosome complement. After the G-banding pattern was assessed, 2 large submetacentrics in the female karyotype were identified as X chromosomes, and the smallest acrocentric in the male karyotype was the Y chromosome (Fig. 2).

C-heterochromatic blocks were revealed in the pericentric regions of 7 bi-armed autosomes (№ 2, 4–9) and the X chromosomes. The autosome 6 has the largest C-block (Fig. 1b). Slightly visible C-blocks were observed in the pericentric regions of certain acrocentrics. The dot-like Y chromosome was C-positive.

**Figure 3.**
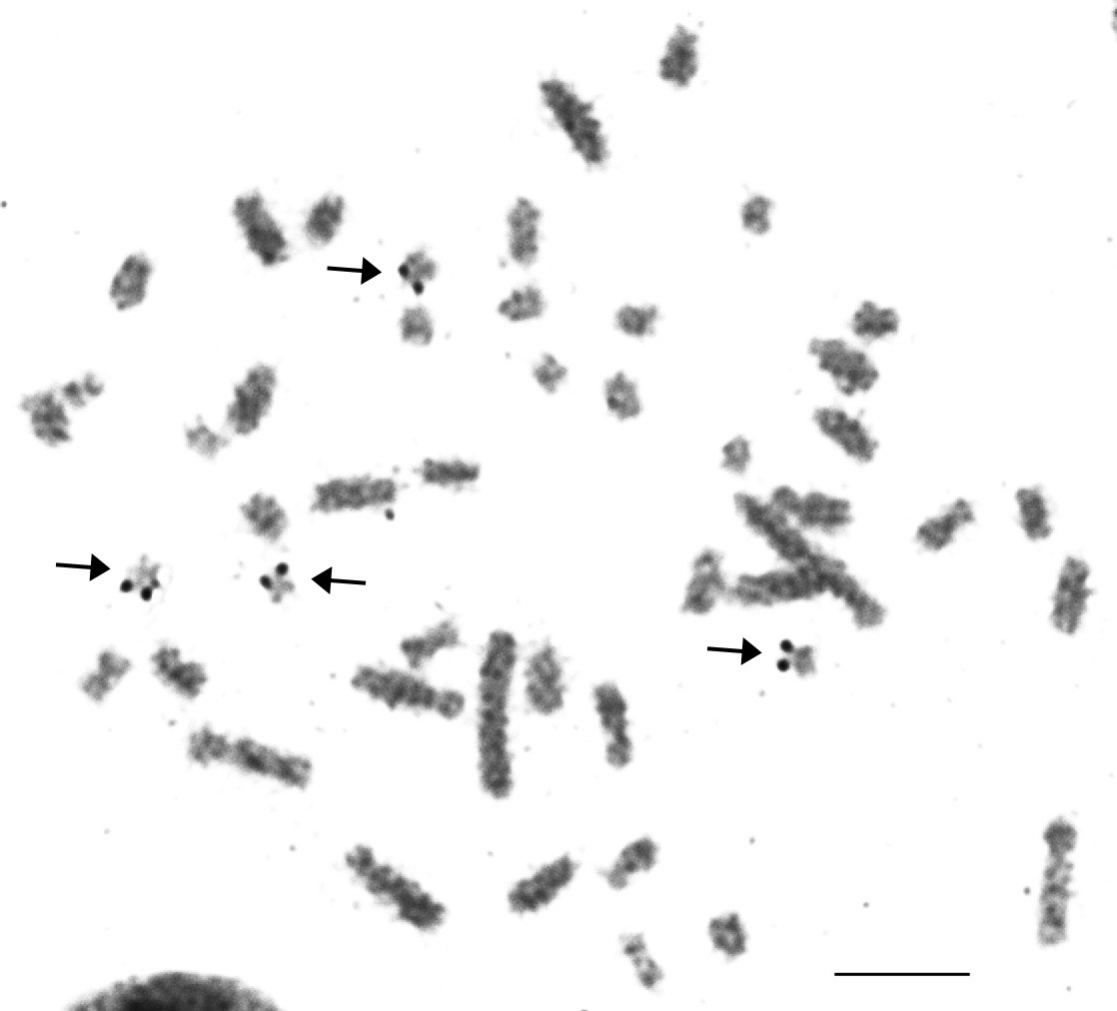
Ag-stained female karyotype of the lesser gymnure *H.suillusmicrotinus* from northern Vietnam. Black arrows indicate the localizations of NORs. Bar = 10 µm.

By silver nitrate staining the terminal localization of NORs was revealed on p-arms of 2 pairs of small autosomes in the female karyotype (Fig. 3).

**Figure 4.**
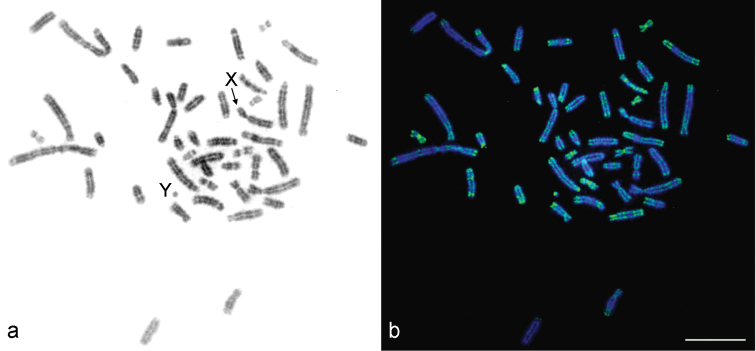
CMA_3_-DAPI stained male karyotype of the lesser gymnure *H.suillusmicrotinus* from northern Vietnam: inverted DAPI**(a)** and CMA_3_-DAPI staining **(b)**. X and Y indicate the sex chromosomes. Bar = 10 µm.

CMA_3_-positive blocks were seen in the telomeric and pericentric regions of most bi-armed and acrocentric chromosomes (Fig. 4). However, the intensity of a signal varied between chromosomes – the brightest fluorescent signals were detected on bi-armed autosome pairs 6, 8, and 9. The Y chromosome had a CMA_3_-positive signal.

**Figure 5.**
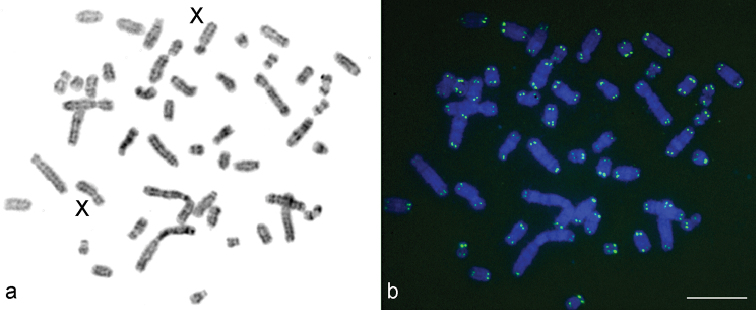
FISH on G-banded chromosomes **(a)** of the female of the lesser gymnure *H.suillusmicrotinus* from northern Vietnam using a fluorescein-conjugated PNA probe **(b)**. Chromosomes counterstained with DAPI (an image is inverted). XX – the sex chromosomes. Bar = 10 µm.

The hybridization with the telomeric DNA (telDNA) probe revealed distinct signals at the telomeres of all chromosomes in the female karyotype (Fig. 5). No interstitial positive signals of telDNA were detected.

### Karyotype of gymnure from southern Vietnam (*Hylomys* sp.)

**Figure 6.**
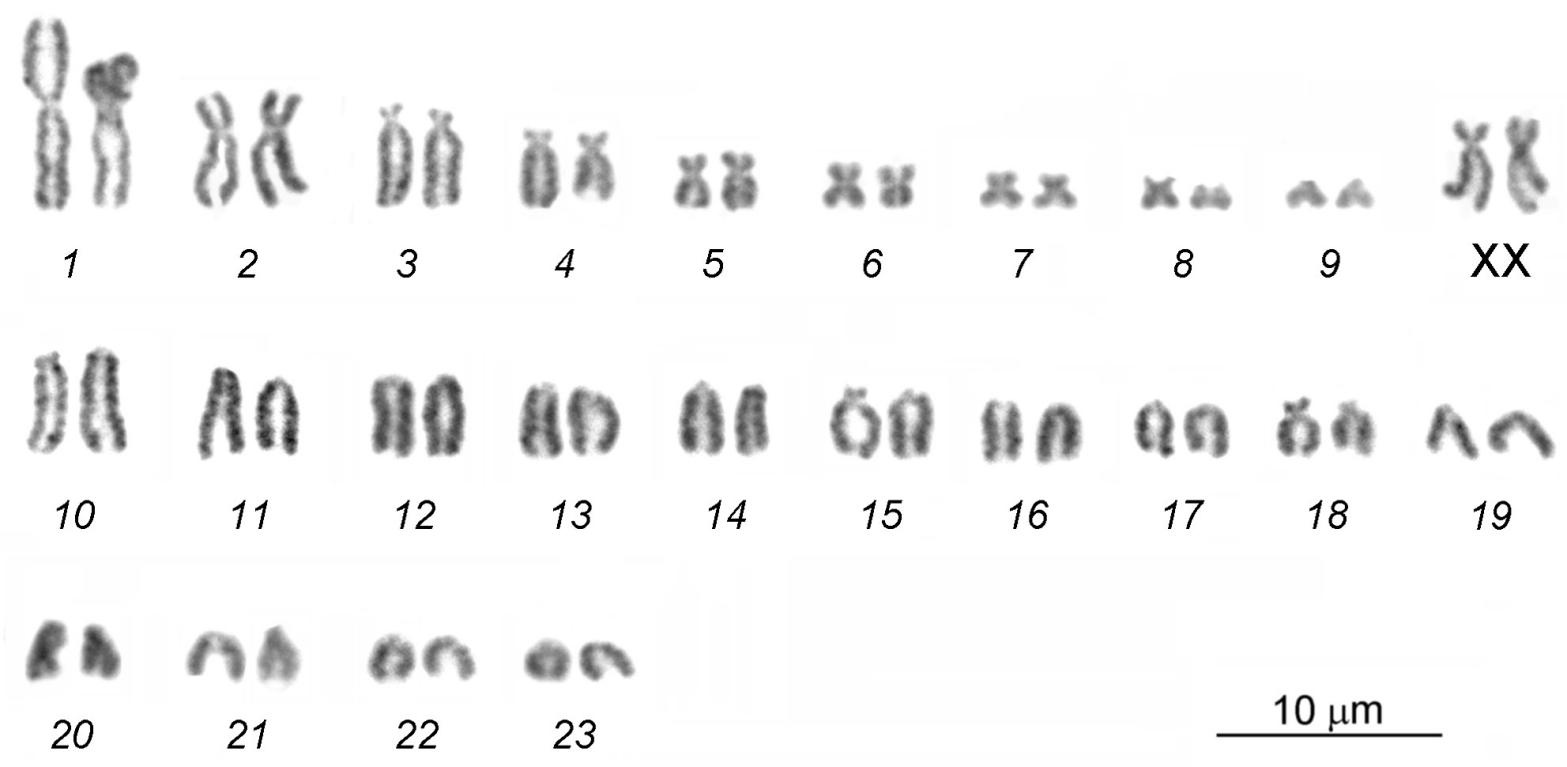
Routine stained female karyotype of the lesser gymnure *Hylomys* sp. from southern Vietnam. 2*n*=48, NF*a*=64.

The karyotype of the female had the same number of chromosomes and chromosome arms as the karyotypes above: 2*n*=48, NF*a*=64 (Fig. 6). The quality of the chromosome suspension that was established in the field was too poor to perform differential stains. The silver nitrate staining revealed that at least one pair of small submetacentrics is bearing Ag-NORs. Nevertheless, the chromosome set appeared to be similar to the gymnure karyotypes from northern Vietnam. It consists of 10 pairs of bi-armed chromosomes including medium-sized submetacentric X chromosomes and 14 pairs of acrocentrics (at least 2 pairs had short arms).

## Discussion

Karyotypes of several hundred mammalian species have been described in the several decades since the development of various methods for characterizing the chromosome sets (O’Brien et al. 2006). Until recently, the order Erinaceomorpha was unevenly studied cytogenetically. More attention was paid to the karyotypes of hedgehogs, whereas gymnures were omitted from even conventional cytogenetic analyses. Three previously studied gymnure species demonstrated large variations in diploid chromosome numbers (Rickart 2003, Ye et al. 2006, Li et al. 2008), implicating karyotype reorganization in the speciation of this clade.

The karyotypes of lesser gymnures from northern and southern Vietnam that we studied had 2*n*=48 and NF*a*=64. These data were first reported for the genus *Hylomys* and *H.suillus*-group. The karyotype structure and chromosome number differed from those of 3 karyotyped gymnure species: *P.truei*, *N.sinensis*, and *N.hainanensis* (Rickart 2003, Ye et al. 2006, Li et al. 2008). Their karyotypes have a smaller 2*n* (40, 32, and 32) and include up to 11 submeta/metacentric and 4-8 subtelo/telocentric autosomal pairs, whereas the studied *Hylomys* karyotypes consisted of 9 submeta/metacentric and 14 pairs of acrocentric autosomes. There are no data on the variation in C-heterochromatin between these 3 species, because only routine staining was applied to them, and *N.sinensis* and *N.hainanensis* were treated by G-banding (Rickart 2003, Ye et al. 2006, Li et al. 2008).

The sex chromosomes of all studied gymnure species, including *Hylomys*, had similar morphologies – the X chromosome is a mid-sized submetacentric, and the Y chromosome is the smallest acrocentric. However, notably, the Y chromosome in *N.hainanensis* is a small metacentric.

Based on an unpublished mtDNA analysis (Bannikova et al. in prep.), our specimens from northern Vietnam (ZMMU S-193936 and ZMMU S-199642) belong to the *H.s.microtinus* lineage. The specimen from southern Vietnam (ZIN 101915) clustered with the distinct genetic lineage of *Hylomys* sp. from southern Vietnam in Bannikova et al. (2014). The large genetic distances (~17% for cyt*b*) imply that this undescribed form of the lesser gymnure should be treated as a separate species, which appears to be the sister group to all taxa of *H.suillus*-group from Southeast Asia (Bannikova et al. 2014).

The diploid number in all spiny hedgehogs (Erinaceidae) studied so far appears to be 2*n*=48 (Orlov and Bulatova 1983, Reumer and Meylan 1986, Hübner et al. 1991, O’Brien et al. 2006), whereas the intrageneric variation in 2*n* in gymnures (Galericidae) is much higher (2*n*=32, 40, 48). This fact might also reflect the deep diversification of gymnures compared with spiny hedgehogs, as demonstrated by the molecular phylogenetic analysis (Bannikova et al. 2014). Despite the similarity in chromosome numbers, there is some variation in karyotype structure (the amount of C-heterochromatin, NORs) in hedgehog species (Gropp 1969, Sokolov et al. 1991). Our results showed that the 2*n* of *Hylomys* individuals from Vietnam are similar to that in spiny hedgehogs; however, this difference might have resulted from the disparate ways of karyotypic evolution in these two divergent groups.

In conclusion, here we have provided the first karyotype description of 2 potential species – *Hylomyssuillusmicrotinus* and *Hylomys* sp.–which are distributed throughout the northern and southern parts of Vietnam, respectively. A detailed characterization of the karyotype of *Hylomys* sp. from southern Vietnam by different chromosome staining is needed to provide a comprehensive comparison between these 2 forms.
